# Fingolimod does not prevent syndecan-4 shedding from the endothelial glycocalyx in a cultured human umbilical vein endothelial cell model of vascular injury

**DOI:** 10.1186/s40635-022-00462-7

**Published:** 2022-08-18

**Authors:** Elissa M. Milford, Lara Meital, Anna Kuballa, Michael C. Reade, Fraser D. Russell

**Affiliations:** 1 Faculty of Medicine, University of Queensland, Herston, QLD Australia; 2 Intensive Care Unit, Royal Brisbane and Women’s Hospital, Butterfield St., Herston, QLD Australia; 3 School of Health and Behavioural Sciences, University of the Sunshine Coast, Maroochydore, QLD Australia; 4 Centre for Bioinnovation, University of the Sunshine Coast, Maroochydore, QLD Australia; 5 Joint Health Command, Australian Defence Force, Canberra, ACT Australia

**Keywords:** Fingolimod, FTY720, Glycocalyx, Endothelium, Cell culture, Syndecan-1, Syndecan-4, Fresh frozen plasma, Sphingosine 1-phosphate, Human umbilical vein endothelial cells

## Abstract

**Background:**

Shedding of the endothelial glycocalyx (EG) is associated with poor outcomes in a range of conditions including sepsis. Fresh frozen plasma (FFP) restores the damaged EG to baseline thickness, however the mechanism for this effect is unknown, and some components of FFP have adverse effects unrelated to the EG. There is some limited evidence that sphingosine-1-phosphate (S1P) within FFP restores the EG by activating the endothelial cell S1P receptor 1 (S1PR_1_). However, there are disadvantages to using S1P clinically as an EG restorative therapy. A potential alternative is the S1PR agonist fingolimod (FTY720). The aim of this study was to assess whether FTY720 prevents EG shedding in injured cultured human umbilical vein endothelial cells.

**Methods:**

Shedding of the EG was induced in cultured human umbilical vein endothelial cells (HUVECs) by exposure to adrenaline, TNF-α and H_2_O_2_. The cells were then assigned to one of six conditions for 4 h: uninjured and untreated, injured and untreated, injured and treated with FTY720 with and without the S1PR_1_ inhibitor W146, and injured and treated with 25% FFP with and without W146. Syndecan-4, a component of the EG, was measured in cell supernatants, and syndecan-4 and thrombomodulin mRNA expression was quantitated in cell lysates.

**Results:**

The injury resulted in a 2.1-fold increase in syndecan-4 (*p* < 0.001), consistent with EG shedding. Syndecan-4 and thrombomodulin mRNA expression was increased (*p* < 0.001) and decreased (*p* < 0.05), respectively, by the injury. Syndecan-4 shedding was not affected by treatment with FTY720, whereas FFP attenuated syndecan-4 shedding back to baseline levels in the injured cells and this was unaffected by W146. Neither treatment affected syndecan-4 or thrombomodulin mRNA expression.

**Conclusions:**

FTY720 did not prevent syndecan-4 shedding from the EG in the HUVEC model of endothelial injury, suggesting that activation of S1PR does not prevent EG damage. FFP prevented syndecan-4 shedding from the EG via a mechanism that was independent of S1PR_1_ and upregulation of SDC-4 production. Further studies to examine whether FTY720 or another S1PR agonist might have EG-protective effects under different conditions are warranted, as are investigations seeking the mechanism of EG protection conferred by FFP in this experimental model.

## Introduction

The endothelial glycocalyx (EG) is a 0.2–5 μm layer of glycoproteins, proteoglycans, glycosoaminoglycans and plasma proteins that lines the vascular endothelium [1]. It is a key regulator of endothelial function, with an important role in vascular permeability, cell–vessel interactions, blood rheology, mechanotransduction, inflammation, coagulation and fibrinolysis [1].

Damage to the EG causes intraluminal shedding of its components. This occurs in critical illness including trauma and sepsis and is correlated with poor outcomes [2]. While it is still unclear whether this association is a causal relationship, there is interest in developing therapies that repair the EG as no current treatment specifically targets the restoration of endothelial integrity [2]. The only resuscitation fluid shown to repair the EG is fresh frozen plasma (FFP); in pre-clinical studies it restores the damaged EG to baseline thickness [3]. However, FFP contains over 1,000 proteins and numerous soluble mediators [4]. Which of these are responsible for EG protection are unknown, and there are indications that some components of FFP have detrimental effects [5].

Sphingosine 1-phosphate (S1P) regulates the cardiovascular, immune, nervous and endothelial systems via the five S1P-specific G protein-coupled receptors (S1PR_1–5_) [6]. Of these, only S1PR_1-3_ are expressed on endothelial cells [7]. A potential mechanism of FFP’s EG-protective effect is the activation of the endothelial cell S1PR_1_. Activation of S1PR_1_ on endothelial cells by S1P in vitro inhibits matrix-metalloproteinases (MMPs), preventing EG shedding [8], and in vivo, attenuates endothelial hyperpermeability in animal models of haemorrhagic shock [9]. FFP contains S1P [10], and there is some limited evidence that S1P is the mediator responsible for FFP’s EG-protective effect. In a cell culture model, FFP stored for 5 days no longer prevented EG damage, and this was associated with a decrease in S1P concentration. The supplementation of 5-day-old FFP with S1P restored its EG-protective effect back to the same level as one-day-old FFP [11].

There are some disadvantages to using S1P clinically as an EG restorative therapy. Firstly, S1P is not approved for therapeutic use and hence translation into practice would take considerably longer than an approved agent due to the necessary regulatory processes. Secondly, because S1P is a non-selective agonist it also activates endothelially expressed S1PR_2_, which has an opposing effect to S1PR_1_. Activation of S1PR_1_ promotes vascular integrity, whereas S1PR_2_ is critical for regulating the endothelial response to inflammatory stimuli causing increases in vascular permeability, and a pro-adhesive and procoagulant phenotype [12]. The balance between S1PR_1_ and S1PR_2_ expression on the endothelium determines the phenotypic response to an inflammatory stimulus [12]. Potentially, selective activation of S1PR_1_, or blockade of S1PR_2_, will result in a greater endothelial and EG-protective effect than non-selective activation of S1PR_1_ and S1PR_2_.

A potential candidate drug that addresses both these issues is FTY720 (fingolimod), an S1PR agonist with high affinity for S1PR_1_ but approximately tenfold less affinity for S1PR_3_ and very little for S1PR_2_ [13, 14]. FTY720 is approved for the treatment of multiple sclerosis [13], and has a half-life in vivo of 6 to 9 days [15]. The main adverse effects of FTY720 include mild and transient bradycardia and atrioventricular block that can be attenuated by atropine and β_2_-adrenoceptor agonists [13, 16]. In animal models of sepsis, trauma, and myocardial infarction, FTY720 reduces vascular permeability, ischaemia–reperfusion injury in solid organs, and lung injury [9, 17–20]. However, its effects specifically on the EG are unknown. The aim of this study was to assess whether FTY720 prevents EG shedding in injured cultured endothelial cells. We hypothesised that FTY720 would be at least as efficacious as FFP.

## Methods

### Human umbilical vein endothelial cell culture

Human umbilical vein endothelial cells (HUVECs) were purchased from Lonza (Lonza, Walkersville, USA). Cells were seeded onto fibronectin-coated 75-cm2 flasks using complete media (EGM-2 BulletKit, Lonza, Walkersville, USA) and grown to confluence in a humidified, 5% CO2 incubator at 37 °C. Cells were lifted from the flasks using 0.25% trypsin/EDTA solution (Gibco, Canada) [21]. Media were replaced daily and cells from passages 2–5 were used for assays. Cells were seeded onto 24-well plates and grown to confluence.

### Experimental design

Cells were assigned to one of six groups: no injury or treatment (control group), injury only, injury treated with FTY720 with and without W146, and injury treated with FFP with and without W146.

As FTY720 is a pro-drug requiring phosphorylation to its active form by EC [22], cells assigned to the FTY720 groups were exposed to 50 ng/mL of FTY720 (Sigma-Aldrich, Macquarie Park, Australia) for 24 h prior to injury exposure to allow sufficient activation of FTY720. At the same time, all cells were exposed to serum-depleted media for 24 h prior to injury to prevent the higher protein environment from confounding the experimental protocol.

A 5 mM solution of the S1PR_1_ antagonist W146 (Tocris Bioscience, Bristol, UK) was prepared in 100 mM sodium hydroxide (Merck, Massachusetts, USA) and stored at – 80 °C. Cells assigned to W146 groups were exposed to 10 $\upmu$M W146 or vehicle (sodium hydroxide) for 30 min prior to injury as described by Zeng et al. [8].

Cells assigned to the injury groups were then exposed to 1.0 nM adrenaline (Aspen Pharmacare, St Leonards, Australia), 10 ng/mL TNF-α (Abcam, Melbourne, Australia), and 100 $\upmu$M H_2_O_2_ for 4 h in a 5% CO_2_ incubator at 37 °C. Cells assigned to the FFP groups were exposed to 25% FFP (Precision BioLogic Inc., Dartmouth, Canada). All cells were incubated with 0.2 U/mL heparin (Pfizer, Sydney, Australia) to prevent fibrin formation given the use of FFP.

**Fig. 1:**
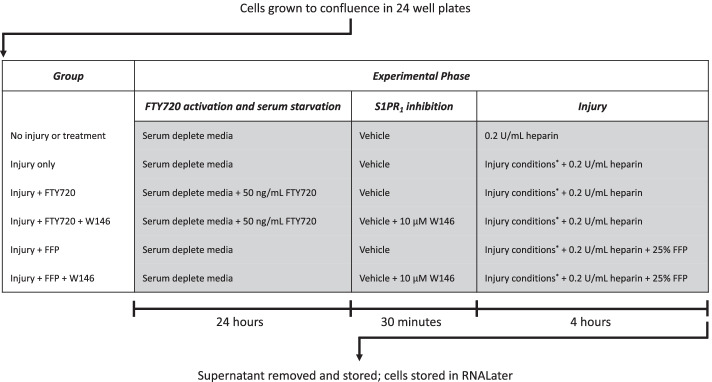
Experimental protocol. Asterisk indicates injury conditions: 1.0 nM adrenaline, 10 ng/mL TNF-α, and 100 μM H2O2 in a 5% CO_2_ incubator at 37 °C

After 4-h exposure to injury and treatment conditions, supernatant was removed, centrifuged for 5 min at 10,000×*g* and stored at − 80 °C for later analysis. Cells were stored in RNALater (Qiagen, Clayton, Australia) at 4 °C for 24 h then at − 20 °C prior to qPCR analysis. The experimental protocol is illustrated in Fig. 1.

### Syndecan-1, syndecan-4, and thrombomodulin analysis in HUVEC supernatant

Syndecan-1 (SDC-1), syndecan-4 (SDC-4), and thrombomodulin (TM) are components of the EG and were measured in HUVEC supernatant using commercially available enzyme-linked immunosorbent assay (ELISA) kits according to manufacturer instructions (SDC-1, Diaclone, France; SDC-4, Sigma-Aldrich, Macquarie Park, Australia; TM, R&D Systems Europe, UK).

### qPCR analysis

Isolation of mRNA was performed using an Isolate II RNA mini kit (Meridian Bioscience, Memphis, USA) and reverse transcription was then performed with a SensiFAST cDNA synthesis kit (Meridian Bioscience, Memphis, USA). Gene expression levels were measured by quantitative PCR using a SensiMix SYBR No-Rox kit (Meridian Bioscience, Memphis, USA) and a Rotor-Gene Q thermal cycler (Qiagen, Clayton, Australia) according to the manufacturer’s protocol. Primers for glyceraldehyde-3-phosphate dehydrogenase (GAPDH), SDC-4, TM, and glypican-1 (GP-1) were as described by Liu et al. [23] and Yang et al. [24]. All samples were analysed in duplicate, and standards were analysed in triplicate. Reference samples and no-template controls were included in each run and no contamination was observed. Quantification of relative gene expression was derived from the relative standard curve method with copy numbers normalised to GAPDH for all samples.

### Statistical analysis

Statistical analysis was performed with GraphPad Prism 9 (GraphPad Software, Inc). Statistical significance was inferred at *p* < 0.05. All data are expressed as mean ± SEM for *n* = 5 experiments. As we had a small sample size, normality was assessed using the Shapiro–Wilk test and for all data this did not demonstrate evidence of non-normality (*p* > 0.05). Visual inspection of the QQ plots was also consistent with normally distributed data. The Brown–Forsythe test for all data was consistent with approximately equal variances between groups (*p* > 0.05). We therefore chose to use a parametric test. An analysis of variance was performed with the Tukey’s multiple comparisons test.

## Results

### Syndecan-1, syndecan-4, and thrombomodulin analysis in HUVEC supernatant

**Fig. 2:**
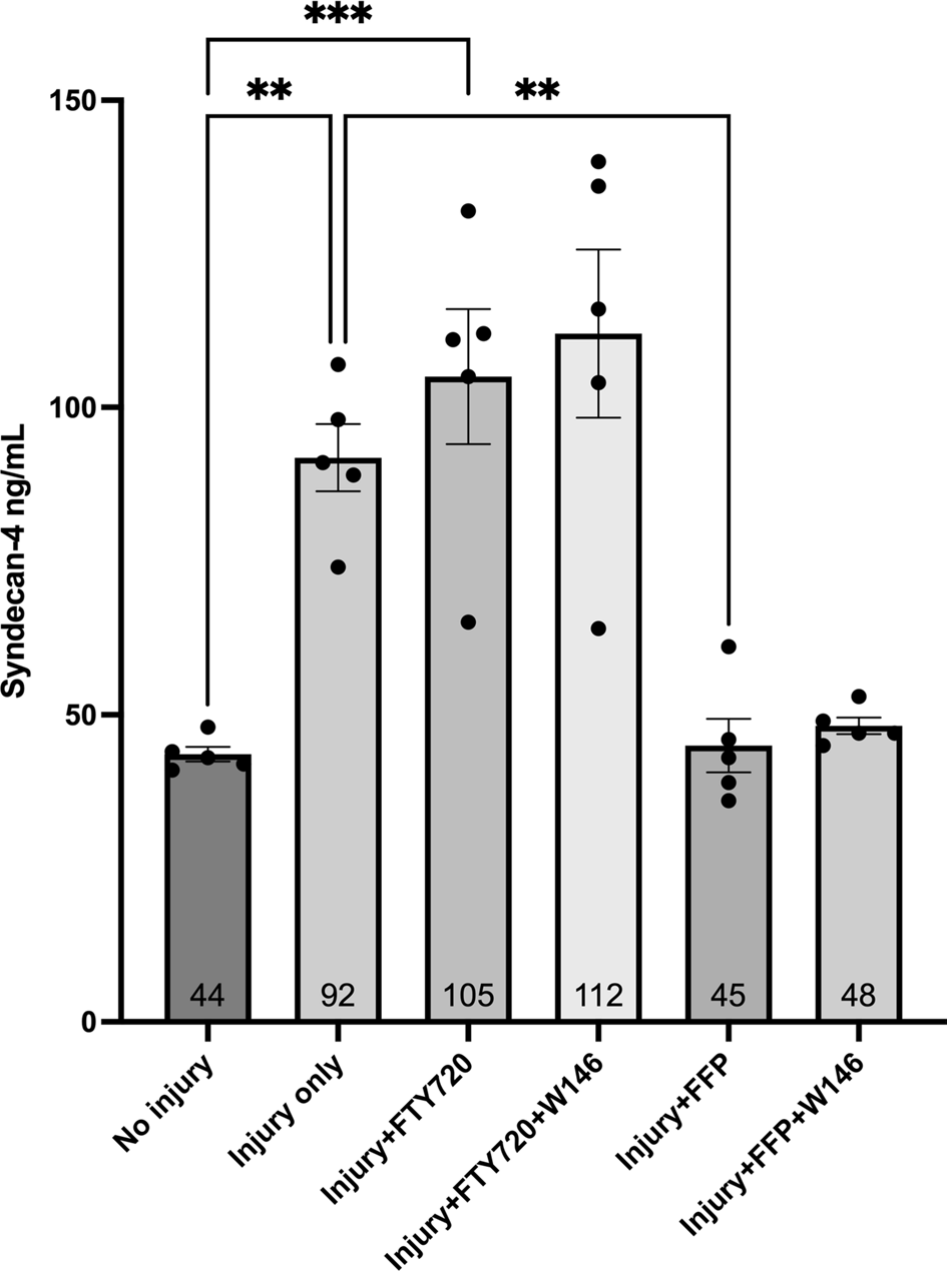
Syndecan-4 (SDC-4) concentration in cell culture supernatant. The effect of treatment with FTY720 or fresh frozen plasma (FFP) with and without 10 $\upmu$M of the sphingosine 1-phosphate receptor 1 (S1PR_1_) antagonist W146 on SDC-4 shedding from injured cultured human umbilical vein endothelial cells (HUVECs). **p < 0.01, ***p < 0.001; *N* = 5 for each group

Cell injury caused a 2.1-fold increase in the amount of SDC-4 in HUVEC supernatants (Fig. 2) compared to the uninjured control (*p* < 0.01). Pre-exposure to FTY720 did not reduce the amount of SDC-4 shed into the supernatant. SDC-4 levels returned to uninjured baseline levels in the FFP treated groups. The inhibitory effect of FFP was not reversed by the addition of W146. The concentration of SDC-4 in the FFP sample (no cells) was 58 ng/mL.

SDC-1 and TM levels in HUVEC supernatants were below the minimum detectable levels for all groups, except for the FFP samples which were 30 ng/mL and 0.9 ng/mL, respectively.

### qPCR analysis

**Fig. 3:**
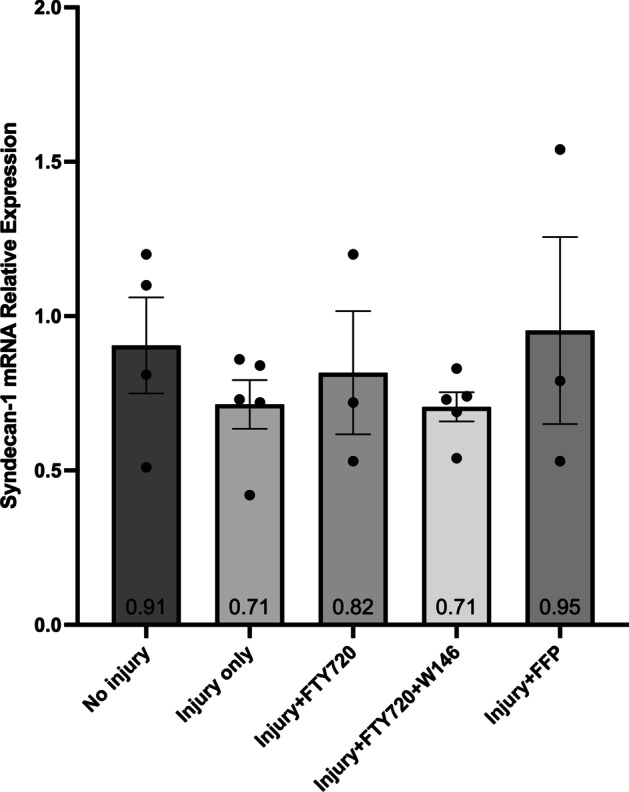
Relative expression of syndecan-1 (SDC-1) mRNA. The effect of treatment with FTY720, with and without 10 $\upmu$M of the sphingosine 1-phosphate receptor 1 (S1PR_1_) antagonist W146, or fresh frozen plasma (FFP) on the relative expression of SDC-1 mRNA from injured cultured human umbilical vein endothelial cells (HUVECs)

**Fig. 4:**
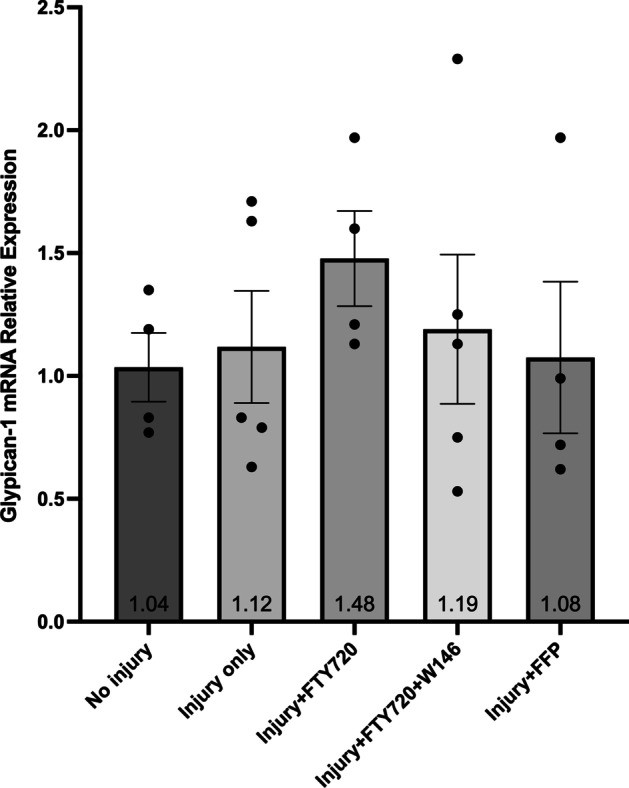
Relative expression of glypican-1 (GP-1) mRNA. The effect of treatment with FTY720, with and without 10 $\upmu$ M of the sphingosine 1-phosphate receptor 1 (S1PR_1_) antagonist W146, or fresh frozen plasma (FFP) on the relative expression of GP-1 mRNA from injured cultured human umbilical vein endothelial cells (HUVECs)

**Fig. 5:**
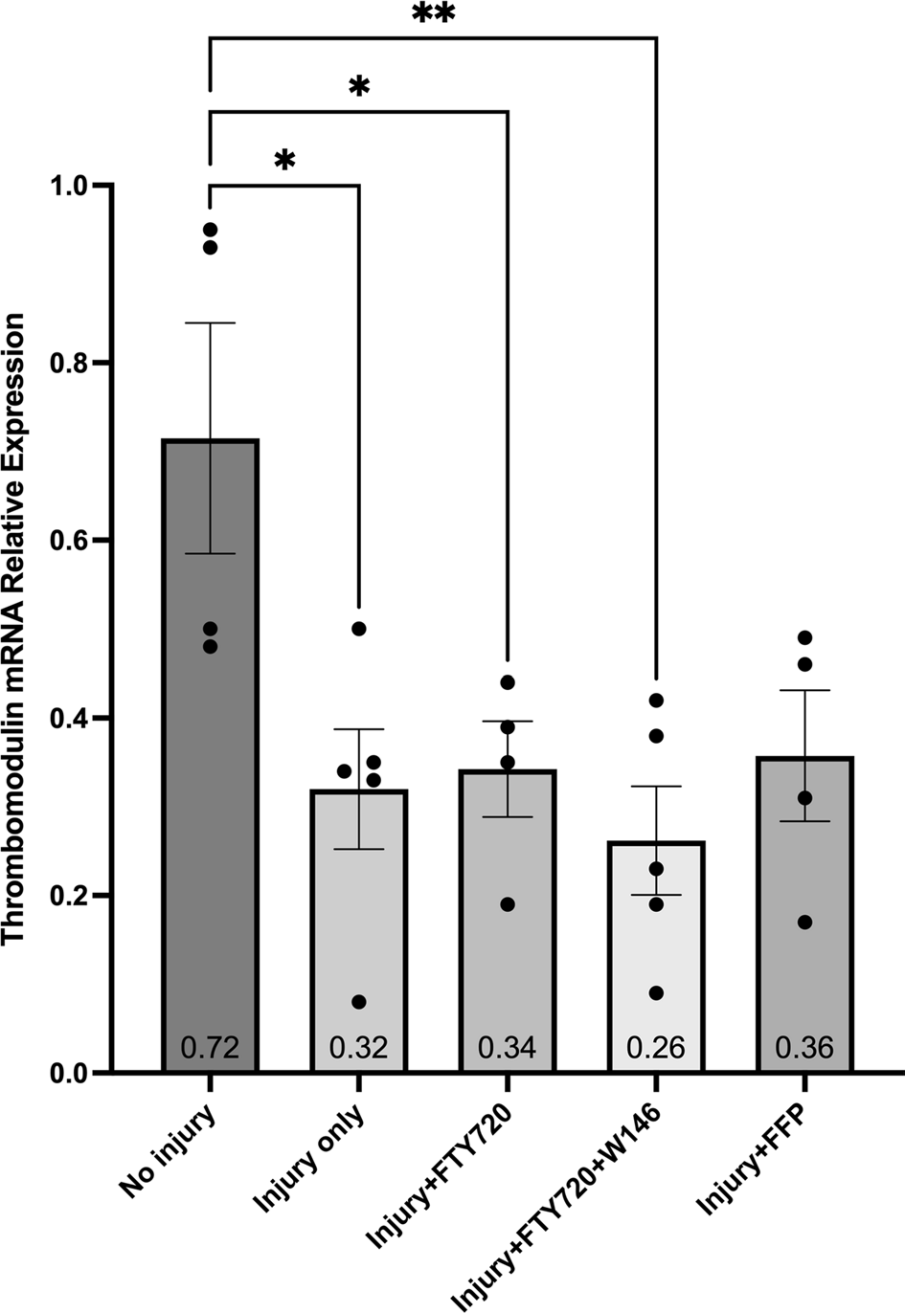
Relative expression of thrombomodulin (TM) mRNA. The effect of treatment with FTY720, with and without 10 $\upmu$M of the sphingosine 1-phosphate receptor 1 (S1PR_1_) antagonist W146, or fresh frozen plasma (FFP) on the relative expression of TM mRNA from injured cultured human umbilical vein endothelial cells (HUVECs). *p < 0.05, **p < 0.01, *N* = 5 for each group

**Fig. 6:**
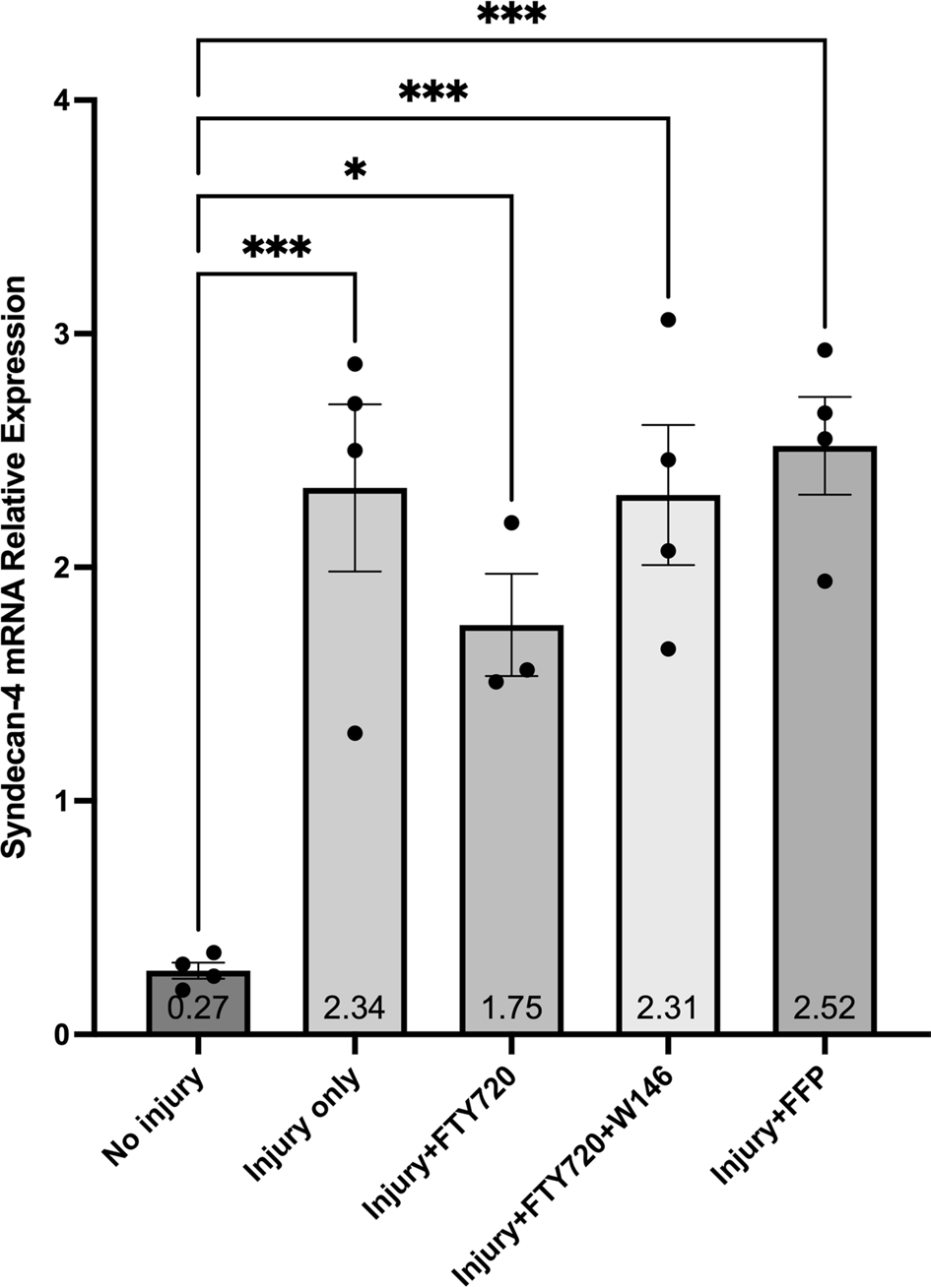
Relative expression of syndecan-4 (SDC-4) mRNA. The effect of treatment with FTY720, with and without 10 $\upmu$ M of the sphingosine 1-phosphate receptor 1 (S1PR_1_) antagonist W146, or fresh frozen plasma on the relative expression of SDC-4 mRNA from injured cultured human umbilical vein endothelial cells (HUVECs). *p < 0.05, **p < 0.01, ***p < 0.001; *N* = 5 for each group

There were no significant differences between any groups for the relative expression of SDC-1 (Fig. 3) or GP-1 (Fig. 4) mRNA suggesting there was no effect of injury or treatment on SDC-1 or GP-1 mRNA expression. Cell injury decreased TM mRNA expression (Fig. 5), but there was no effect of FFP or FTY720 treatment. Cell injury increased SDC-4 mRNA expression (Fig. 6), but there was also no effect of FFP or FTY720 treatment. A non-significant trend for decreased expression of SDC-4 in the injury and FTY720 group was observed.

## Discussion

### Main findings

FTY720 did not prevent SDC-4 shedding in this study of injured cultured HUVECs, suggesting that FTY720 does not prevent EG shedding in injured endothelial cells. Following exposure to EG shedding conditions for 4 h, the concentration of SDC-4 in supernatants was significantly higher than in uninjured cells. However, the concentrations of SDC-4 in the culture supernatants were similar for cells exposed to FTY720 or vehicle. SDC-4 is one of the structural backbones of the EG and its presence in the supernatant is consistent with damage to the EG [1]. FTY720 also did not appear to stimulate the increased production of EG components in the context of a damaged EG, with no increase in the relative expression of SDC-1, SDC-4, TM, or GP-1 mRNA compared to the injured, non-treated control cells. Together, these data do not support EG-protective effects of FTY720 at the concentration used in this study. This contrasts with cells treated with FFP, where the SDC-4 supernatant levels were similar to the uninjured control cells.

### Possible reasons for lack of efficacy of FTY720

There are several potential reasons that FTY720 did not protect the EG in this study. Firstly, it is possible that in addition to any direct agonist effect, FTY720 was also acting as a functional antagonist at the S1PR_1_. It is known to cause initial activation but then degradation of S1PR_1_ in lymphocytes, which is its mechanism of action in the treatment of multiple sclerosis. This is in contrast to the natural ligand S1P which has an EC_50_ of 302 nM for S1PR_1_ degradation compared to 0.34 nM for FTY720 [25]. However, FTY720 is thought to have a different effect on endothelial S1P receptors with persistent signalling by FTY720 at S1PR_1_ after internalisation in cultured HUVECs [26, 27]. Cells were exposed to FTY720 for 24 h prior to injury to allow sufficient time for the cells to phosphorylate it into its active form [22] and to maximise any potential effect, so it is likely that the receptors would have been internalised by the time of injury exposure. There was a non-significant trend of higher SDC-4 levels in the FTY720 groups, and slightly higher again with the addition of an S1PR_1_ antagonist, compared to the injury only group. While this would be consistent with functional antagonism, testing a shorter exposure duration with pre-activated FTY720 or testing a different S1PR agonist may provide clarification. FTY720 is the first S1PR agonist to be approved for therapeutic use, however there are newer S1PR agonists in development that do not cause receptor desensitisation [27].

Inadequate dosing is another possible cause of lack of efficacy in this study. We chose a dose (50 ng/mL or 160 nM) that is used clinically and well above the threshold known to improve vascular permeability, but it is possible this is too low to achieve EG protection. Clinically, maximal lymphopenia is seen at trough levels of 10 ng/mL, and optimal efficacy for immunosuppression in transplantation is around 50 ng/mL [16]. Concentrations as low as 10 nM improve vascular permeability in HUVECs while high doses, up to 1000 nM, are known to cause adverse effects including an increase in pulmonary vascular permeability [28]. The natural ligand S1P achieves EG protection in cultured HUVECs at a concentration of 187 nM, while 87 nM does not, suggesting there is a steep dose–response curve between around 100 to 200 nM [8]. Using the membrane binding assay GTPγS, the EC_50_ at S1PR_1_ has been reported as between 0.4 to 1.2 nM for S1P and a similar 0.3 to 2 nM for FTY720 [13, 14]. This suggests there may be a similar dose–response curve for FTY720 as S1P in terms of EG protection, although it is unknown whether the GTPγS EC_50_, which reflects membrane receptor binding, corresponds to the downstream effect of EG protection. It is therefore possible the dose of 160 nM was inadequate to offer full EG protection and this does warrant further exploration. It is unlikely that there was not enough active form of FTY720 in the cell culture supernatant. Cells were pre-treated with FTY720 for 24 h prior to being injured. Activation to the phosphorylated active form occurs within 3 h by endothelial cells [16], but 24 h was chosen for this study to maximise activation. The half-life of FTY720 in vivo is 6 to 9 days with primarily hepatic metabolism, so it is very unlikely levels dropped within 24 h [15].

A less likely but possible reason that FTY720 does not prevent EG shedding is because of its S1PR_1_ and, to a lesser extent with tenfold less affinity, S1PR_3_ selectivity [13, 14]. S1PR_1_ is thought to mediate most of the effects of S1P on the endothelium including S1P’s EG-protective effect and has a functional antagonistic relationship to S1PR_2_ [12]. Less is known about the role of S1PR_3_ on the endothelium, but it does appear to have a similar function to S1PR_2_ [7]. There may be unknown interactions between the three endothelial S1PRs that result in an EG-protective effect with activation of S1PR_1-3_ by S1P but not from activation of S1PR_1_ and S1PR_3_ by FTY720.

### Limitations of the model

The conditions associated with EG shedding are diverse, and include ischaemia and reperfusion, sepsis, trauma, atherosclerosis, and diabetes [29]. These conditions act via a diverse range of intermediary mediators in complex and not well understood pathways, but likely converge on a common pathway resulting in enzymatic cleavage of EG components from the endothelium by sheddases including matrix metalloproteases, A disintegrin, heparanase, and hyaluronidases [29, 30]. Given their complexity, simulating these processes in vitro is difficult. In this study, we took a multi-faceted approach to the injury and used a combination of three agents (adrenaline, TNF-α, and H_2_O_2_) at concentrations that have previously been demonstrated to induce EG shedding in vitro [31, 32] and are increased in vivo as part of the inflammatory response in sepsis and trauma clinically [31, 33].

Exposure to these agents for 4 h induced EG shedding as evidenced by significantly higher SDC-4 levels in the supernatant of the injury group compared to non-injured controls. SDC-4 is one of the four sub-types of trans-membrane syndecans that form the main structural elements of the EG. Most clinical studies have measured SDC-1 as a marker of EG shedding as the predominant syndecan shed into the blood of critically unwell patients is SDC-1 and -3, and SDC-4 and SDC-2 levels are not significantly elevated compared to healthy controls [34]. SDC-1 cell surface expression is upregulated in response to shear stress [11, 35, 36], and this downregulates the expression of SDC-4 via cell signalling pathways [37], but not vice versa. Instead, SDC-4 upregulation appears to be a compensatory response to the decreased expression of SDC-1 [37]. Therefore, when endothelial cells are cultured in static conditions, the predominant syndecan subtype expressed is SDC-3 and -4, with relatively low expression of SDC-1 and -2 [38]. There are also other differences between endothelial cells cultured under flow compared to static conditions. There is a linear relationship between shear stress and the rate of EG growth [36, 39, 40]. Higher shear stress conditions result in increased expression of hyaluronan [35, 36], intracellular adhesion molecule 1 (ICAM-1) [36, 40], and von Willebrand Factor [36]. The net effect is a thicker EG, reduced lymphocyte adhesion, increased platelet adhesion, and reduced permeability, in endothelium exposed to high compared to low shear stress [36].

Given these differences, the clinical relevance of a statically cultured HUVEC model of EG injury and treatment is unclear and may be a limitation of this study. However, although not well characterised, SDC-1 and SDC-4 appear to have similar, but not identical, roles in regulating vascular endothelial function [35, 37, 41]. Most relevant to the prevention of EG shedding of critical illness, the MMP-mediated mechanism for SDC-1 and SDC-4 shedding is the same [8, 38], and the EG-protective effects of FFP are seen clinically [42], in animal models [3], and in static [43] and flow [11] cultured HUVECs. Statically cultured HUVECs also express S1PR_1_ [8]. Therefore, the differences between the flow and static cultured EG are unlikely to impact on the assessment of a therapy that targets the S1PR_1_ receptor to prevent MMP-mediated shedding of EG components. However, it is still possible that FTY720 has a differential effect on SDC-1 and SDC-4 shedding through an unknown mechanism. The effect of FTY720 on the shedding of SDC-1 and other EG components should be assessed in a flow cultured model to investigate this further.

### Fresh frozen plasma

The results of this study are consistent with the well-established EG-protective effects of FFP [44]. The mechanism for this effect is unknown, but has been speculated to involve S1P [11, 44] or another component such as fibrinogen [45], antithrombin-III [46], or heparanase-2 [47]. S1P has previously been shown to mediate its EG-protective action via S1PR_1_ [8]. In this study, the blockade of S1PR_1_ did not alter the efficacy of FFP, suggesting that the EG-protective properties of FFP are not mediated by this receptor as has been suggested by previous studies where S1P-depleted FFP regained its EG-protective properties with S1P supplementation, as measured by SDC-1 shedding [11]. It may be that S1P in FFP mediates its effects by preventing cleavage of SDC-1 and not SDC-4 from the endothelium. FFP did not increase the relative expression of SDC-4 mRNA in the injured cells, suggesting the mechanism is due to prevention of shedding rather than an increase in production of EG. Further research is required to further define the role of S1P and S1PR_1_ in the mechanism of FFPs EG-protective effect, and then whether specifically targeting this pathway will achieve the benefits of FFP while avoiding its adverse effects.

## Conclusion

In conclusion, this study suggests that FTY720 does not prevent EG shedding in injured cultured HUVECs. The previously reported attenuation in endothelial cell hyperpermeability by FTY720 [9, 17–20] may be due to a different mechanism than EG shedding such as promotion of endothelial cell adherens junction assembly [16], a reduction in apoptosis and oxidative stress mediated by intracellular signalling modulation [20, 48, 49], and a reduction in leukocyte recruitment [50, 51]. However, due to its limitations this study cannot exclude the possibility that FTY720 or another S1PR agonist has EG-protective effects under different conditions. Further work is required to investigate the influence of timing of exposure, dose, inflammatory stimulus, culture conditions, cell type, and effects on different EG components. The other significant finding of this study was that FFP prevented EG shedding, consistent with previous evidence [3]. However, this does not appear to be mediated by activation of S1PR_1_ or by upregulation of SDC-4 production. The search for the mechanism(s) of FFPs protective effect is ongoing. Furthermore, it should be noted that while EG shedding is associated with poor outcomes in critical illness, there is no evidence yet that protection and repair of the EG improves clinical outcomes. The identification of a targeted EG therapy will allow this hypothesis to be tested.

## Data Availability

The datasets used and/or analysed during the current study are available from the corresponding author on reasonable request.

## Abbreviations

EG: Endothelial glycocalyx
FFP: Fresh frozen plasma
S1P: Sphingosine 1-phosphate
S1PR: Sphingosine 1-phosphate receptor
MMP: Matrix-metalloproteases
HUVEC: Human umbilical vein endothelial cells
SDC-1: Syndecan-1
SDC-4: Syndecan-4
TM: Thrombomodulin
ELISA: Enzyme-linked immunosorbent assay
GP-1: Glypican-1
ICAM-1: Intracellular adhesion molecule 1
